# Long-Term Survival Following Cardiac Arrest Without Implantable Defibrillator Protection in a Hypertrophic Cardiomyopathy Patient

**DOI:** 10.4021/cr46w

**Published:** 2011-05-20

**Authors:** Mustafa Cetin, Ozgul Ucar, Alper Canbay, Zehra Guven Cetin, Hulya Cicekcioglu, Erdem Diker

**Affiliations:** a Ataturk Chest Disease and Chest Surgery Education and Research Hospital, Department of Cardiology, Sanatoryum Caddesi, 06280, Ankara, Turkey; b Ankara Numune Education and Research Hospital, Department of Cardiology, Talatpasa Bulvari, 06110, Ankara, Turkey; c Medicana International Ankara Hospital, Department of Cardiology, Sogutozu Mahallesi, 2165 Sokak, No: 6, Soguou, Ankara, Turkey

**Keywords:** Hypertrophic cardiomyopathy, Sudden cardiac death, Dilated cardiomyopathy, Atrioventricular block

## Abstract

Hypertrophic cardiomyopathy (HCM) is the most common cause of sudden cardiac death in young people. Implantable cardioverter defibrillator (ICD) is the optimal therapy in patients with HCM, both for primary or secondary prevention of sudden death. Left ventricular systolic function in HCM is usually normal. However, in few patients, HCM has been reported to progress to a state that is characterized by left ventricular dilation and systolic dysfunction, resembling dilated cardiomyopathy (DCM). Although arrhythmias are common in HCM, advanced or complete atrioventricular block (AV) is very rare. This case report describes a HCM patient who progressed to DCM with advanced AV block and survived 31 years following cardiac arrest without ICD protection.

## Introduction

Hypertrophic cardiomyopathy (HCM) is the most common genetically inherited cardiac disorder with an estimated prevalence of 1 in 500 in the general population [1]. The diagnosis of HCM is based on the echocardiographic appearance of left ventricular maximum wall thickness ≥ 15 mm, in the absence of any other cause capable of producing such hypertrophy [2]. The disease is characterized by marked heterogeneity with respect to clinical manifestations, natural history and prognosis. Sudden cardiac death (SCD), its most important complication, is reported to occur with an annual incidence of 2% to 4% in tertiary referral centers [3, 4]. Although ventricular arrhythmias are the most common cause of SCD, atrial fibrillation, atrioventricular (AV) block and rapid AV conduction via an accessory pathway have been suggested to be the other reasons [5].

In this case report, we describe a HCM patient who had been diagnosed 31 years ago following cardiac arrest. The patient later progressed to dilated cardiomyopathy (DCM) and presented to the emergency service because of syncope with advanced AV block.

## Case Report

**Figure 1:**
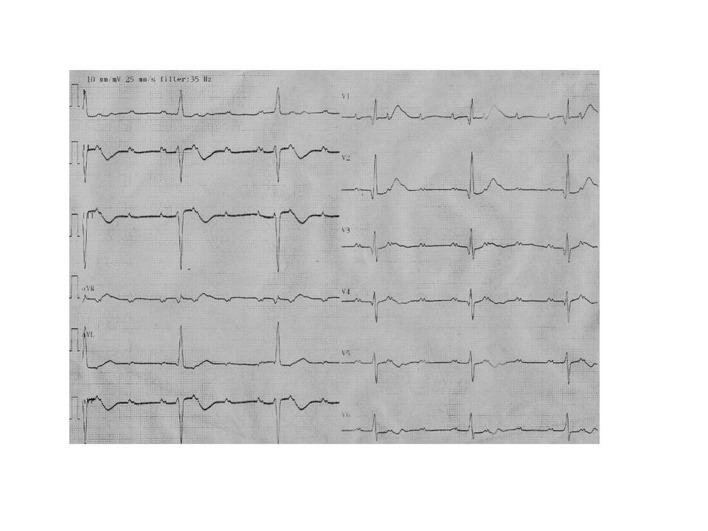
Twelve-lead ECG at the time of admission to the emergency service showing advanced AV block.

**Figure 2:**
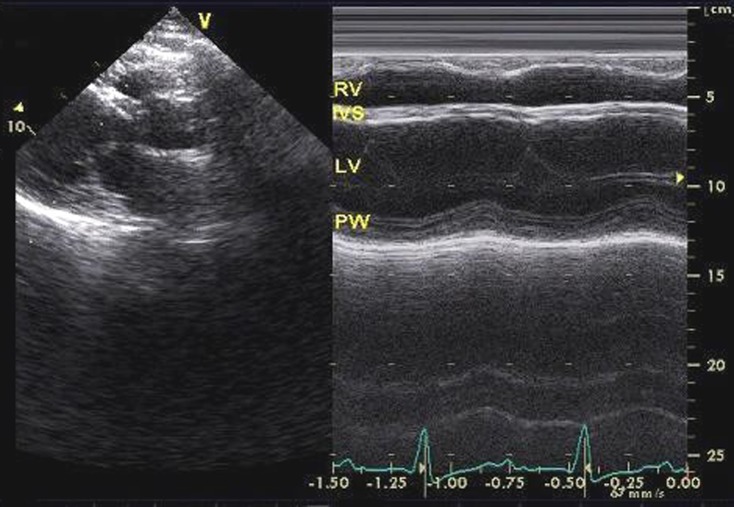
M-mode transthoracic echocardiogram from the parasternal long axis view demonstrating left ventricular dilatation and systolic dysfunction (RV: Right ventricle, IVS: Interventricular septum, LV: Left ventricle, PW: Posterior wall).

**Figure 3:**
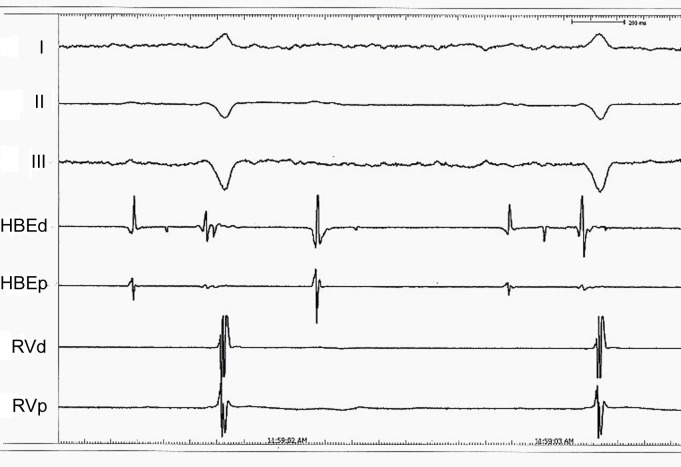
Electrophysiological study showing infra-His 2 : 1 AV block.

A forty-year-old Caucasian man was admitted to the emergency service with the complaints of syncope and progressive dyspnea of six months’ duration. From his history it was learned that he was diagnosed HCM following cardiac arrest 31 years ago. He neither had regular controls, nor took any medications. His blood pressure was measured as 100/60 mmHg and his pulse rate was 35 bpm. His cardiac and respiratory system examinations were normal, except bradycardia in auscultation. On his twelve-lead electrocardiography heart rate was 32 beats/min and electrocardiography indicated advanced AV block (Fig. 1). Transthoracic echocardiography was performed; diffuse hypokinesia of the left ventricle was observed and left ventricular end-diastolic diameter was measured as 6.1 cm (Fig. 2). Left ventricular ejection fraction was detected as 35%. Coronary angiography revealed normal epicardial coronary arteries. Electrophysiologic study was performed at the same session; A-H and H-V intervals were measured as 130 ms and 110 ms, respectively and infrahissian 2 : 1 AV block was demonstrated (Fig. 3). Sustained monomorphic ventricular tachycardia was induced by programmed stimulation via right ventricular apex. Biventricular pacemaker defibrillator was implanted to the patient with the diagnosis of HCM which progressed to DCM with advanced AV block. Family screening revealed non-obstructive HCM in his mother and two siblings.

## Discussion

The greatest SCD risk in HCM patients is the history of cardiac arrest or sustained ventricular tachycardia and in these patients ICD implantation is insistently recommended [6, 7]. However, the longest reported survival after cardiac arrest with or without ICD protection is 30 years [8] and in these patients the annual appropriate shock rate is 11% [9]. In contrast to the circumstance in coronary artery disease, the arrhythmogenic substrate in HCM may remain intermittently dormant. Therefore, initial clinical recognition of high risk status and ICD implantation should be performed many years before the unpredictable occurrence of potentially lethal ventricular arrhythmias that require intervention.

Left ventricular dilation and systolic impairment may be seen in 5% of the HCM patients [6]. Progression to DCM has been reported up to 18% in patients with ‘cardiac myosin binding protein C’ mutation [10]. A study with 248 HCM patients reported that half of the patients who have progressed to DCM had family history of HCM and 25% of them had family history of SCD [11]. No difference was detected in aspect of the cardiac mortality among the patients with and without left ventricular dilation in this study. In our case, the point that shows the importance of genetic factors in the progression process to DCM is the existence of three other family members with HCM.

Although supraventricular and ventricular tachycardias are common in HCM, advanced or complete AV block is very rare [12]. The pathophysiology of AV block in HCM is not known. However, myocardial ischemia, autonomic dysfunction, or an abnormal vascular response in HCM may be one of the underlying mechanisms for AV block [13]. Some patients with HCM may also experience recurrent syncopal attacks due to torsades de pointes following AV block [14]. Doven et al. reported three HCM patients with abnormal His-Purkinje conduction and complete AV block with attacks of syncope or cardiopulmonary arrest [15]. Therefore, advanced or complete AV block should always be kept in mind in the differential diagnosis of syncope and cardiopulmonary arrest in patients with HCM.

In our case, 31 years of cardiac event-free survival following a cardiac arrest without ICD protection in a HCM patient is the most important aspect. Progression to DCM and association with advanced AV block are the other interesting points.
